# Renal flare as a biomarker of incident and progressive chronic kidney disease in patients with lupus nephritis

**DOI:** 10.1186/ar3979

**Published:** 2012-09-27

**Authors:** S Parikh, A McKinley, BH Rovin

**Affiliations:** 1 Wexner Medical Center at Ohio State University, Columbus, OH, USA

## Introduction

Flares of lupus nephritis (LN) cause acute kidney injury (AKI), even if serum creatinine (SCr) does not increase. Although classic forms of AKI, such as ischemia, have long been thought to heal without long-term sequelae, it is now clear that AKI events predispose patients to chronic kidney disease (CKD). It was therefore postulated that renal flare (RF) in LN patients promotes CKD.

## Methods

To determine whether RF frequency and duration can be used as markers of new CKD or progression of established CKD, we correlated RF with starting and ending SCr levels in the Ohio SLE Study (OSS) cohort.

## Results

New-onset CKD occurred in 12/41 patients over a median follow-up of 4.5 years. The CKD group had more RF events than the non-CKD group: 31 (2.59/patient) versus 17 (0.59/patient), respectively, and spent more time in RF (Table 1). Only 8% of the CKD group versus 59% of the non-CKD group had no RF. In OSS patients with established CKD, those who progressed had more RF events than nonprogressors: 13 (1.63/patient) versus 2 (0.29/patient), respectively. In the nonprogressor group 71% had no RF, compared with 37.5% of progressors. Progressors had a significant change in SCr over the study period (*P *= 0.0078). Differences in number of RF and RF duration were not significant between the two groups but tended to be higher in the progressors (Table 2).

**Table 1:** 

	No CKD (*n *= 29)	New CKD (*n *= 12)	*P *value
Age	31.3	34.3	NS
Male (%)	14	0	-
African American (%)	28	50	-
Mean starting SCr (mg/dl)	0.85 ± 0.15	0.75 ± 0.15	NS
Mean end SCr (mg/dl)	0.83 ± 0.12	1.78 ± 1.95	0.0001
Median time in flare (months)	0	20	0.0003
Mean new RF/year	0.14	0.72	0.0001
Median time in renal health (months)	52	30.4	0.0038

**Table 2:** 

	Nonprogressors (*n *= 7)	Progressors (*n *= 8)	*P *value
Age	39.1	40.5	NS
African American (%)	14	62.5	-
Mean starting SCr (mg/dl)	1.94 ± 0.83	2.15 ± 0.81	NS
Mean end SCr (mg/dl)	1.56 ± 0.87	4.0 ± 2.45	0.0093
Mean new RF per year	0.10	0.41	NS
Median time in renal health (months)	48	25	0.0560

## Conclusion

In patients with LN, the frequency and duration of RF are biomarkers of new CKD and progression of existing CKD. As new LN therapeutic regimens are developed, targeting RF prevention should be an important goal.

